# Ultrasound biomicroscopy in glaucoma assessment

**DOI:** 10.22336/rjo.2021.24

**Published:** 2021

**Authors:** Vasile Potop, Valeria Coviltir, Speranţa Schmitzer, Christiana Diana Maria Dragosloveanu, Cătălina Ioana Ionescu, Miruna Gabriela Burcel, Maria Cristina Corbu, Dana Margareta Cornelia Dăscălescu

**Affiliations:** * Ophthalmology Department, “Carol Davila” University of Medicine and Pharmacy, Bucharest, Romania; ** Ophthalmology Department, Clinical Hospital of Ophthalmologic Emergencies, Bucharest, Romania; *** Ophthalmology Department, Oftaclinic Bucharest, Romania

**Keywords:** ultrasound biomicroscopy, primary open angle glaucoma, primary angle closure glaucoma

## Abstract

Ultrasound biomicroscopy (UBM) is an important tool in the diagnosis, evaluation and follow up of glaucoma patients. Even if we are dealing with a primary angle closure glaucoma (PACG) or a primary open angle glaucoma (POAG) patient, the mechanism of angle closure can be revealed by performing an UBM. The device can help differentiate between the two types of glaucoma even in patients with opaque corneas when gonioscopy cannot be performed. Knowing the type of glaucoma is vital, especially regarding an individualized treatment, since each patient is unique and needs to be treated accordingly, in order to prevent glaucomatous optic neuropathy and visual field loss.

**Abbreviations**:

AC = anterior chamber, ICE = iridocorneal endothelial syndrome, IOP = intraocular pressure, NTG = normal tension glaucoma, PACG = primary angle closure glaucoma, PC = posterior chamber, PEX = pseudoexfoliation syndrome, POAG = primary open angle glaucoma, UBM = ultrasound biomicroscopy

## Introduction

Ultrasonography uses pulses of ultrasonic waves in order to capture images of deep structures in the body. The sound waves are directed into the tissue, and are reflected when encountering a different density [**1**]. It typically uses low frequency emitting probes in order to capture images at a greater depth, with the limitation of a lower resolution [**1**-**3**]. Conversely, a high frequency emitting probe leads to images with greater resolution, but cannot investigate deeper tissues [**1**,**4**].

Ultrasound biomicroscopy was described as an ophthalmologic application of ultrasonography more than 15 years ago by the team led by Charles J. Pavlin [**1**]. In ophthalmology, ultrasounds are usually performed within the 10 MHz range, yet UBM is a special type of ultrasound that can use frequencies up to the 50-100 MHz range [**1**,**2**,**5**].

The advantage is that a higher frequency probe can provide highly detailed images, but it cannot view deeper, posterior structures of the eye [**1**,**4**,**6**]. Therefore, UBM is a suitable tool to investigate the anterior segment of the eye, and the best tool for analyzing the structures posterior to the iris, even in eyes with opaque media. When using conventional probes, it is difficult to maintain a good balance between resolution and depth, but when using a 50 MHz probe we can obtain images of structures that are about 4 mm deep, at a resolution of about 40 microns [**2**,**3**,**7**-**9**]. After the information is captured, the device uses the signal-processing box in order to transmit the signal to the computer [**5**,**6**,**10**].

The major difference between the B scan ultrasonography and ultrabiomicroscopy is that the latter needs the transducer to be immersed to provide high-resolution images. We can use either saline solution or methylcellulose 1% [**10**]. Air cannot provide the same transmission of the ultrasound waves, so we must use a coupling agent [**4**]. The technique of ultrasonography, on the other hand, is easier to perform, but leads to images of a lower resolution [**4**].

UBM allows the visualization of all the anterior segment structures: cornea, sclera, anterior chamber, lens, zonules, ciliary body and processes, iris, scleral spur, and ciliary sulcus [**11**,**12**].

Using the linear, 16 mm width UBM probe, placed at 90 degrees on the surface of the eye, it is possible to visualize, in one scan, all the above-mentioned structures and to take accurate measurements [**5**,**11**]. The clinical applications of UBM can be found in corneal pathology, refractive surgery, glaucoma management and surgical cases, trauma, and can be considered a key investigation in anterior segment tumors, cataract, lens and IOL placement, iris, and ciliary body pathology [**11**,**13**-**15**].

## Ultrabiomicroscopic aspect of physiological ocular features

UBM can evaluate the structures of the eye at microscopic resolution [**14**,**16**]. The most anterior structure is the cornea, seen as a curved hyperreflective line. We can evaluate its components, thickness, and shape [**6**]. Due to its superficial placement, we can even measure the thickness of its components, such as the epithelium, besides the global thickness, with a high-resolution transducer [**6**,**14**]. The anterior chamber (AC) is encountered posterior to the cornea and anterior to the iris [**6**]. UBM imaging provides information regarding the shape, content, angle, and can be used to measure its dimensions. Normally, AC depth should be about 2.5-3.0 mm, but with significant variations [**5**]. The iris can be found posterior to the AC, with a flat, hyperechogenic aspect, connected laterally to the ciliary body and scleral spur [**6**,**17**]. UBM is probably the most important investigation in the pathology of the iris and ciliary body [**4**,**17**]. We can examine its shape, position, insertion, and rapports to other ocular structures [**17**]. In patients with a pupillary block, the iris can have a convex appearance [**10**,**18**].

Another major structure visualized by UBM is the lens. The examination provides important information regarding its configuration, position, and anteroposterior diameter. This investigation should be performed preoperatively, in a thorough manner, as it can reveal an enlarged lens that generates phacomorphic glaucoma, a shallow AC, or a displaced lens, both of which may influence the outcome of cataract surgery [**19**]. Most laterally is the sclera, seen as a highly echogenic structure. Its thickest component is the scleral spur. Higher frequency probes (in the range of 100 MHz) have been developed, enabling the visualization of Schlemm’s canal [**8**,**19**,**20**].

## Indications of UBM

UBM can be used in several disorders of the anterior ocular segment, including in cases of extensive corneal opacities, which represent a contraindication for anterior segment OCT [**21**]. It can help as a diagnostic procedure in eyelid and anterior segment (cornea, conjunctiva, sclera, iris, ciliary body) pathology. It is a vital tool in evaluating anterior segment cysts or tumors and their diagnosis, evolution, and treatment [**22**]. In glaucoma patients, UBM can help differentiate many glaucoma types, evaluate the anatomy of the angle or the position and shape of the iris [**23**,**24**].

Moreover, it can be used preoperatively, as an investigation tool in difficult cases of cataract or glaucoma surgery, or postoperatively, in order to evaluate the filtration bleb, the position of the aqueous shunts, the position of the iris, ciliary body and lens [**4**].

## Contraindications of UBM

UBM is contraindicated in patients with ocular penetrating and perforating injuries, in the early postoperative period, with infections of the ocular surface or eyelids, and in patients who do not cooperate [**4**].

## UBM and glaucoma

The first applications of UBM in glaucoma have been described by C. J. Pavlin in 1992, when the team led by him defined the normal range for several anterior segment parameters, such as AC depth, trabecular iris angle, or scleral and iris thickness [**25**]. Furthermore, UBM is recognized in the latest edition of the European Glaucoma Society Guideline as a valuable imaging tool to measure the dimensions of anterior segment structures [**26**] and to correctly classify glaucoma patients: primary closed angle cases, including congenital, juvenile, or adult onset, secondary closed angle, or even certain types of secondary open angle glaucoma. In this paper, we examined several subtypes of glaucoma, whose characteristics can be identified by UBM [**14**,**17**,**19**].

UBM also provides information for patients with secondary angle closure glaucoma [**19**]. It could identify the mechanism of glaucoma in patients with iridociliary tumors, cysts located in the anterior segment, ciliary body, or pars plana, microspherophakia or lens subluxation. UBM also provides an accurate assessment of iris/ ciliary body cysts, evaluates their rapports and precise localization. As a rule, a cyst appears as a well delineated lesion with a thin wall, without a solid component [**9**,**21**,**27**]. 

Gonioscopy is the first intention procedure for AC angle evaluation, but it is a procedure that requires good cooperation with the patient, an intact clear cornea and conjunctiva, as well as a skilled examiner [**17**,**21**]. Gonioscopy may provide important information when it can be performed, but it is subjective and there are limitations in the absence of transparent media, especially in patients with corneal edema, secondary to elevated intraocular pressure (IOP). In these cases, UBM is the first choice in order to provide the necessary information.

## UBM and primary congenital glaucoma

In primary congenital glaucoma, UBM can produce valuable data regarding the morphology of the anterior segment structures, even in patients with opaque corneas. The investigation can be used in most patients, including young children. The most frequently encountered anomaly is the lack of the iris crypts, but UBM assessment can detect changes such a large corneal diameter, reduced corneal thickness, a shallow AC or a large AC angle, ciliary body abnormalities or a reduced PC [**9**,**28**-**33**]. Barkan’s membrane, a thin, hyperechogenic structure, overlaying the AC angle, may also be visible by UBM examination [**9**].

## UBM and primary angle closure glaucoma

UBM is an important instrument in evaluating PACG patients. Regardless of the angle closure mechanism, understanding iris and ciliary body configurations is essential in the workup of any PACG patient [**17**]. UBM can reveal the anatomy of the entire anterior segment, which in PACG is crowded, usually due to an anteriorly positioned lens [**34**]. Studies on patients evaluated in a scotopic environment revealed the changes that appear in the anterior segment in low light conditions [**9**,**24**]. This technique could also help in the follow up of PACG patients, as it allows for precise measurements of anterior segment structures and for following changes in these structures, in time [**34**].

Using UBM, the exact site of angle closure can be identified. Studies revealed that these sites could be either at the iris pupillary margin in cases of pupillary block, the lens in phacomorphic glaucoma, the ciliary body in plateau iris configuration, or posterior to the lens, in malignant glaucoma [**17**,**20**,**24**]. A thin ciliary body could explain an anteriorly positioned lens that determines a shallow AC and a narrow angle [**20**]. Knowledge of the ocular morphology, in each case, allows for a correct, personalized treatment plan [**17**]. 

## UBM and pupillary block 

In cases of pupillary block, UBM identifies the exact configuration of the lens-iris diaphragm and the convex appearance of the iris associated with a shallow AC [**17**,**19**]. In these patients, due to the mechanical obstruction, the aqueous humor cannot pass from the posterior chamber to the anterior chamber, the iris being, thus, pushed anteriorly [**17**]. Following aqueous humor buildup in the posterior chamber, the pressure in it becomes higher than the pressure in the AC [**9**,**35**].

Performing laser iridotomy flattens the iris and relieves the pressure difference between the anterior and the posterior chamber [**19**,**24**,**35**].

## UBM and plateau iris configuration 

Plateau iris configuration is a particular anatomic variant, in which the iris and ciliary body are larger or displaced anteriorly and, thus, could determine angle closure in young individuals [**36**-**38**]. The diagnosis is made during a routine examination or in the presence or glaucoma symptoms [**24**]. Even if the slit lamp examination is normal at first glance, gonioscopy reveals a narrow angle and a double hump configuration of the iris [**37**,**38**]. Following diagnosis, treatment response is often poor: patients are usually non-responsive to laser peripheral iridotomy, even if the procedure is properly performed and the iridotomy is patent [**37**,**39**].

UBM can assist in the identification of the exact anatomical rapport between the iris and the ciliary body, and it may identify a possible associated ciliary block. We could either encounter an anterior angulation of the iris, a flat iris, anteriorly positioned ciliary processes, a short or thick iris root or iridotrabecular contact that associate a normal depth of the anterior chamber centrally [**9**,**19**,**37**,**38**,**40**].

## UBM and lens related glaucoma

Abnormalities in lens shape and position could determine an anterior displacement of the iris with secondary glaucoma. Causes could include trauma, zonular laxity, or certain forms of cataract [**3**,**7**,**24**]. Most commonly, UBM can document all these changes, which lead to impaired aqueous humor outflow and, subsequently, IOP raise.

Phacomorphic glaucoma is considered a secondary angle closure glaucoma (SACG), brought by an advanced cataract, which can cause the antero-posterior diameter of the lens to increase, leading to pupillary block, trabecular meshwork obstruction and impairing aqueous humor elimination. Thus, it may cause a quick IOP elevation that affects the optic nerve head and, in time, leads to glaucomatous optic neuropathy [**41**]. The same mechanism has been reported in young individuals with a large clear lens that obstructs the angle, but the phacomorphic component is hidden due to the lack of cataract [**42**].

The phacomorphic glaucoma determines IOP peaks clinically associated with corneal edema and pain. Therefore, gonioscopy examination is difficult to perform and provides limited information regarding angle structures. UBM is a useful tool for the assessment of these cases [**41**]. It is a noninvasive test that provides a complete evaluation of the anterior segment structures, including AC depth, the lens shape, and the iridocrystalline diaphragm position in rapport to adjacent structures, revealing the mechanism of glaucoma [**41**]. IOP lowering medication followed by lens extraction through phacoemulsification is the treatment plan of choice [**41**].

## UBM and malignant glaucoma

This type of glaucoma, also known as aqueous misdirection or ciliary block, represents a challenging diagnosis for every ophthalmologist [**17**]. Studies revealed that in patients with malignant glaucoma developed after trabeculectomy, UBM could reveal certain characteristics, including flattening of the anterior chamber, even the presence or absence of fluid in the supraciliary space [**17**,**19**]. The ciliary body is anteriorly rotated and thinner compared to the fellow eye. This anatomical position could be the decisive factor in the development of malignant glaucoma [**43**].

## UBM in Iridocorneal endothelial (ICE) syndrome

This syndrome is defined by the abnormal proliferation of corneal endothelium, which leads to changes in the iris, corneal endothelium and iridocorneal angle that could determine secondary glaucoma [**44**-**46**]. UBM may reveal specific ICE findings, such as corneal edema, iris atrophy, Descemet folds, peripheral anterior synechiae [**9**,**24**]. Since anterior segment OCT and gonioscopy are impossible to perform in patients with corneal edema, UBM remains the only valuable imaging tool [**9**,**24**,**47**].

## UBM and pigmentary glaucoma

In pigment dispersion syndrome, the posterior surface of the iris sheds pigment, which accumulates in different structures of the anterior ocular segment, including the trabeculae. Pigmentary dispersion in the trabecular meshwork leads to trabecular outflow impairment and a raised IOP [**9**,**19**,**47**]. In these patients, we can also encounter iris concavity that may be increased by accommodation. During accommodation, the lens’ antero-posterior diameter increases and the IOP is slightly higher [**19**,**48**]. Laser iridotomy appears to have a positive effect [**48**]. 

## UBM and Pseudoexfoliation syndrome

Pseudoexfoliation syndrome (PEX) is believed to be a systemic condition, with distinctive ocular features, resulting from the deposition of a fibrillary material in the anterior segment of the eye [**49**]. In patients with PEX, UBM shows zonular defects, exfoliating material on the lens capsule, and sometimes a flat AC and a large lens [**9**,**50**,**51**]. Furthermore, UBM can detect nodular deposits on the zonules [**52**].

## UBM and Glaucoma surgery 

Even if we talk about Schlemm’s canal procedures, subconjunctival filtration or suprachoroidal drainage, we need to have accurate information regarding the anatomy of each eye before surgery [**53**,**54**]. 

In cases of patients requiring deep sclerectomy or canaloplasty, UBM can also be helpful in the postoperative evaluation of the filtering bleb and Schlemm’s canal [**55**,**56**]. Therefore, UBM is a vital tool in the guidance and preparation of glaucoma surgery [**19**,**53**,**57**]. Its utility is also found in cases of postoperative complications. Additionally, it allows the measurement of the intrascleral space defined by the trabeculectomy glaucoma surgery [**55**,**56**]. Using UBM, the morphologic changes that appeared after glaucoma surgery can be examined [**53**,**55**].

Trabeculectomy is one of the most important surgery methods in glaucoma and UBM is an important tool in bleb follow up [**58**,**59**]. It allows the functional evaluation of the bleb and its filtration rate and, in case of postoperative complications, it allows the exact localization of the lesion and the development of an appropriate treatment plan. The reflectivity of the sclera and the aspect of the scleral flap represent predicting factors in the bleb outcome [**58**].

Therefore, in these cases, UBM is an important device in association with gonioscopy, anterior segment OCT and slit lamp examination [**21**].

## UBM Advantages

UBM is considered one of the best technologies that visualizes the ciliary body [**21**]. This makes it essential in assessing the angle configuration and the mechanism of closure in a primary angle-closure suspect or glaucoma case [**11**]. It is also vital in the plateau iris configuration and retroiridal processes [**37**]. 

Furthermore, it is the only device that can evaluate the AC in eyes with opaque corneas [**9**]. Also, it is used widely in the preoperative assessment, as it can help the surgeon develop the optimal surgical plan [**11**].

Tumors of the anterior segment can be assessed using UBM, including iris and ciliary body melanomas and their anatomical position and contact [**9**]. Corneal pathology such as scars, dystrophies or degenerations can also benefit from UBM [**9**], without impacting corneal biomechanics [**60**]. Finally, UBM can be easily used in children [**9**].

## UBM Limitations

This imaging technique is a very valuable one, but it is a meticulous process and requires good cooperation with the patient and a skilled operator in order to obtain good quality images. The test works with a coupling agent, usually introduced in an immersion cup. Due to the high risk of contamination, it is contraindicated in trauma to the globe with penetrating scleral or corneal wounds [**11**].

Since the patient is in a supine position, the iris diaphragm is displaced posteriorly and the AC may seem deeper. It is also sensitive to pressure applied to the eye that could modify the rapports within the AC. The angle is documented by superior, inferior, temporal, and nasal parts and not by degrees of an arc as the OCT does, and can only view 1 quadrant at a time [**11**].

## Conclusions

UBM is an important tool in the examination of the anterior segment of the eye. It is significant in patients with opaque corneas, in which conventional examination methods cannot provide many details. 

As for glaucoma patients, UBM can help precisely identify the type of glaucoma, the mechanism of the disease, develop the appropriate treatment plan and follow the patient’s evolution.

**Conflict of Interest statement**

Authors state no conflict of interest.

**Acknowledgments**

All authors had equal participation and contribution to this paper.

**Sources of Funding**

None.

**Disclosures**

None.
